# Supplementary material for: A computational paradigm for dynamic logic-gates in neuronal activity
Source: Front Comput Neurosci. 2014 Apr 29;8:52. doi: 10.3389/fncom.2014.00052 (PMC4010740; doi:10.3389/fncom.2014.00052)
Supplement: Supplementary file 1 [file Presentation1.PDF]

1. Appendix: Materials and methods

1.1. Culture preparation

Cortical neurons were obtained from newborn rats (Sprague -Dawley) within 48 h after birth using mechanical and enzymatic procedures (Marom and Shahaf, 2002). All procedures were in accordance with the National Institutes of Health Guide for the Care and Use of Laboratory Animals and Bar-Ilan University Guidelines for the Use and Care of Laboratory Animals in Research and were approved and supervised by the Institutional Animal Care and Use Committee. The cortical tissue was digested enzymatically with 0.05% trypsin solution in phosphate-buffered saline (Dulbecco's PBS) free of calcium and magnesium, supplemented with 20 mM glucose, at 37°C. Enzyme treatment was terminated with heat-inactivated horse serum, and cells were then mechanically dissociated. The neurons were plated directly onto substrate-integrated multi-electrode arrays (MEAs) and allowed to develop functionally and structurally mature networks over a time period of 2–3 weeks in vitro, prior to the experiments. Variability in the number of cultured days in this range had no effect on the observed results. The number of plated neurons in a typical network was in the order of 1,300,000, covering an area of about 380 mm^2. The preparations were bathed in minimal essential medium (MEM-Earle, Earle's Salt Base without L-Glutamine) supplemented with heat-inactivated horse serum (5%), glutamine (0.5 mM), glucose (20 mM), and gentamicin (10 g/ml), and maintained in an atmosphere of 37°C, 5% CO_2, and 95% air in an incubator as well as during the electrophysiological measurements. All experiments were conducted on cultured cortical neurons that were functionally isolated from their network by a pharmacological block of glutamatergic and GABAergic synapses. Experiments were conducted in the standard growth medium, supplemented with 10 μM CNQX (6-cyano-7-nitroquinoxaline-2,3-dione) and 80 μM APV (amino-5-phosphonovaleric acid). 5 μM Bicuculline was added only in experiments where no inhibitory stimulations were used (**Figures 1,2,3**). This cocktail of synaptic blockers made the spontaneous network activity sparse. At least one hour was allowed for stabilization of the effect.

1.2. Measurements and stimulations

An array of 60 Ti/Au/TiN extracellular electrodes, 30 μm in diameter, and spaced either 200 or 500 μm from each other (Multi-Channel Systems, Reutlingen, Germany) were used. The insulation layer (silicon nitride) was pre-treated with polyethyleneimine (0.01% in 0.1M Borate buffer solution). A commercial setup (MEA2100-2x60-headstage, MEA2100-interface board, MCS, Reutlingen, Germany) for recording and analyzing data from two 60-electrode MEAs was used, with integrated data acquisition from 120 MEA electrodes and 8 additional analog channels, integrated filter amplifier and 3-channel current or voltage stimulus generator (for each 60 electrode array). Mono-phasic square voltage pulses ([100,500] μs, [-900,-100] mV) were applied through extracellular electrodes. Each channel was sampled at a frequency of 50k sample/s. Action potentials were detected on-line by threshold crossing. For each of the recording channels a threshold for spike detection was defined separately, prior to the beginning of the experiment.

1.3. Cell selection

Each logic-gate's node was represented by a stimulation source (source electrode) and a target for the stimulation – the recording electrode (target electrode). These electrodes (source and target) were selected as the ones that evoked well-isolated, well-formed spikes and reliable response with high signal-to-noise ratio. This examination was done with stimulus intensity of -800 mV using 30 repetitions at a rate of 5Hz followed by 1200 repetitions at a rate of 10 Hz.

In experiments where inhibitory stimulations were used (NOT-gate, XOR-gate) Bicuculline was not added to the standard growth medium, hence inhibitory synapses were not blocked. The initial step to identify a pair of electrodes for an inhibitory stimulation was to pinpoint an excitatory node by its source and target electrodes (a stimulation of the source electrode, i, results in a detection of a well isolated spike in the target electrode, j). In the next step, stimulations were given to each one of the 60 extracellular electrodes (electrode k) a few ms prior to the stimulation of the source electrode, i, while the activity of the target electrode, j, was recorded. This procedure was repeated five times. This examination was performed under different time-lags between stimulations of electrode k (k=1 to 60) and the stimulation of the source electrode, i. In the case of an inhibitory stimulation (neuron k inhibits neuron j), a stimulation given to electrode k several ms prior to the stimulation of the source electrode (e.g. less than 7 ms, **Figure S1 A** and **Figure S1 B**) results in no neuronal response recorded by the target electrode, j. When the time-lag between the stimulations of electrode k and the source electrode is relatively long (e.g. 15 ms, **Figure S1 C**), the inhibitory effect gradually disappears, and a spike will be detected in the target electrode.

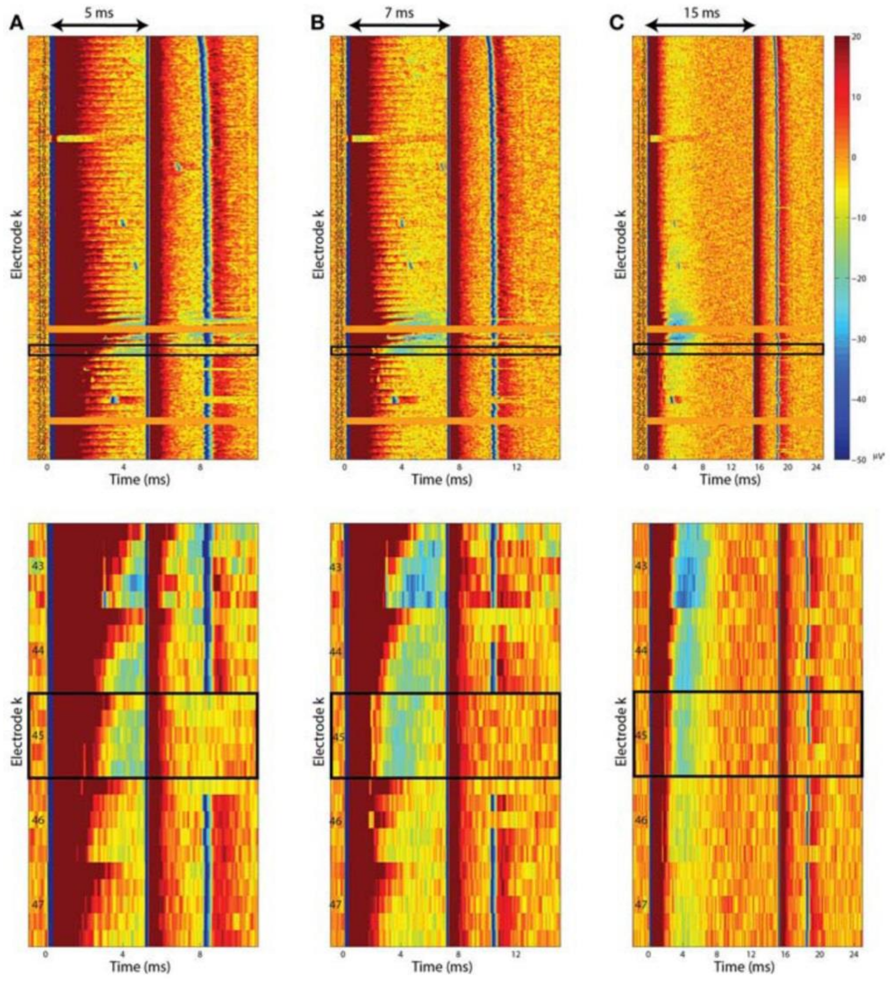

FIGURE S1 | (Color online) Inhibitory stimulations. The voltage recorded from a neuron's target electrode ($j = 42$) is presented in a color scale. Each row is independent from the others, and represents consecutive recordings. Row k represents the effect of an inhibitory stimulation (e.g., $k = 45$, presented enlarged at the lower panel) which precedes the stimulation of the neuron's source electrode ($i = 55$). Stimulations of electrodes $k = 1$–60 are given at time 0 (left dark blue column). Stimulations of the source electrode are given 5 ms **(A)**, 7 ms **(B)**, and 15 ms **(C)** after the stimulation of electrode k (middle dark blue column). The rightmost blue column represents the spikes recorded from the target electrode.

1.4. Stimulation control

A node response was defined as a spike occurring within a typical time window of 2-10 ms following the electrical stimulation. The activity of all source and target electrodes was collected, and entailed stimuli were delivered in accordance to the connectivity of nodes in the logic-gate setup.

Gate connectivity, τ: Conditioned stimulations were enforced on the gate-neurons, embedded within a large-scale network of cortical cells in vitro, following the gate connectivity. Each gate delay is defined as the expected time between the spike and stimulation of two linked neurons; e.g. conditioned to a spike recorded in neuron i, a stimulation will be given to neuron j after K_{ij} (ms). The time-lag between the stimulations of two linked neurons is defined as τ_{ij}. Note that in the case of two neurons $\tau_{ij}=L_i+K_{ij}$, where L_i is the response latency of neuron i.

After an electrical stimulation is given to the output neuron of the gate (neuron E, F, E, G in **Figures 2A, 3A, 4A, 4D**, respectively (Vardi et al., 2013b)), the input neurons (A, B) are simultaneously stimulated again after a fixed delay. The longest path from the input neurons to the output neuron, together with the time-lag between a stimulation applied to the output neuron and the next stimulation of the input neurons, determine the stimulation frequency of all the neurons constituting the gate; e.g. initially in **Figures 2A** (Vardi et al., 2013b) the longest path from the input neurons to the output neuron is 80 ms, and for a 20 ms time-lag between the stimulation applied to the output neuron and the next stimulation of the input neurons the effective stimulation rate of the neuronal gate is ~10 Hz.

AND-gate: Strong stimulations, (-800 mV, 200 μs), which were given to all gate neurons excluding neuron E, result in a reliable neural response. Weak stimulations (-550 mV, 120 μs) were given to neuron E, such that an evoked spike is expected only if the time-lag between two consecutive weak stimulations is short enough. In cases where the time-lag between two consecutive stimulations was shorter than 100 μs (from the end of the first stimulation to the beginning of the consecutive one), a unified long stimulation (-550mV, 280 μs) was applied, to overcome technical limitations. All neurons were stimulated at a rate of 10 Hz.

OR-gate: Strong stimulations (-800 mV, 200 μs), resulting in a reliable neural response, were given to all gate neurons. All neurons were stimulated at a rate of 1 Hz (**Figure 3B** (Vardi et al., 2013b)) or 10 Hz (**Figure 3C** and **Figure 3D** (Vardi et al., 2013b)). Since for each input stimulation neuron F was stimulated twice, its effective stimulation rate in the case of two evoked spikes was 20 Hz (**Figure 3C** and **Figure 3D** (Vardi et al., 2013b)). This higher stimulation rate results in a deterioration of the neuronal response which screens the distinguishing effect of one or two evoked spikes. To prevent this discrepancy, neuron F was stimulated only every second round, such that its effective stimulation rate remains on the average 10 Hz.

NOT-gate: Strong stimulations (-800 mV, 200 μs) were given to all gate neurons, excluding neuron E and result in a reliable neural response. A weaker stimulation (-550 mV, 100 μs) was given to neuron E to enhance the inhibitory effect. All neurons were stimulated at a rate of 1 Hz (**Figure 4B** (Vardi et al., 2013b)) or 10 Hz (**Figure 4C** (Vardi et al., 2013b)).

XOR-gate: Strong stimulations (-800 mV, 200 μs) were given to all gate neurons besides neurons E and F and result in a reliable neural response. Weaker stimulations (-550 mV, 100 μs) were given to neurons E and F to enhance the inhibitory effect. All neurons were stimulated at a rate of 1 Hz. To overcome the low probability to find two inhibitory stimulations in a given culture, the same source and target electrodes were assigned to nodes E and F, and the same inhibitory

electrode was assigned to nodes C and D. These neurons were stimulated in a time-lag of 50 ms, and since the stimulation rate was 1 Hz there were no conflicts in terms of timing (e.g. latency stretching, refractory period, etc.).

1.5. Data analysis

Analyses were performed in a Matlab environment (MathWorks, Natwick, MA, USA). Action potentials were detected by threshold crossing. In the context of this study, no significant difference was observed in the results under threshold crossing or voltage minima for spike detection.

Since only a detection of spike in a certain neuron leads to a conditional stimulation of its linked neuron, there was a need to handle missed stimulations as well as missed evoked spikes. This was handled differently according to the nature of the gate:

Neuronal response latency (**Figure 1** (Vardi et al., 2013b)): In **Figure 1A** (Vardi et al., 2013b) the time-lags between the neuron's evoked spikes and the electrical stimulations are presented. Unconditional stimulations were given at a rate of 10 Hz, indicating that a stimulation is given every 100 ms whether a spike was detected or not. Stimulation instances not resulting in evoked spikes are not shown in the graph. In **Figure 1B** (Vardi et al., 2013b) and **Figure 1C** (Vardi et al., 2013b), the time-lag between the evoked spikes of the input and output neurons are presented. Only instances resulting in an evoked spike of the output neuron are shown.

AND-gate (**Figure 2B** (Vardi et al., 2013b)): Only instances where two stimulations were applied to the output neuron are shown, since one (or zero) stimulation will never generate an evoke spike (see stimulation control, AND-gate).

OR-gate (**Figure 3C** and **Figure 3D** (Vardi et al., 2013b)): Only instances where one or two stimulations were applied to the output neuron are shown. In this case even a single stimulation can evoke a spike (see stimulation control, OR-gate), and marked as '-1'.

NOT-gate (**Figure 4C** (Vardi et al., 2013b)): Only instances where both excitatory and inhibitory stimulations were applied to the output neuron are shown. The probability of an evoked spike of the output neuron is calculated only when the two stimulation types are applied (see stimulation control, NOT-gate).
